# From mice to men: lessons from mutant ataxic mice

**DOI:** 10.1186/2053-8871-1-4

**Published:** 2014-06-16

**Authors:** Jan Cendelin

**Affiliations:** Department of Pathophysiology, Faculty of Medicine in Pilsen, Charles University in Prague, Lidicka 1, 301 66 Plzen, Czech Republic; Biomedical Centre, Faculty of Medicine in Pilsen, Charles University in Prague, Plzen, Czech Republic

**Keywords:** Ataxia, Cerebellum, Neurodegeneration

## Abstract

Ataxic mutant mice can be used to represent models of cerebellar degenerative disorders. They serve for investigation of cerebellar function, pathogenesis of degenerative processes as well as of therapeutic approaches. Lurcher, Hot-foot, Purkinje cell degeneration, Nervous, Staggerer, Weaver, Reeler, and Scrambler mouse models and mouse models of SCA1, SCA2, SCA3, SCA6, SCA7, SCA23, DRPLA, Niemann-Pick disease and Friedreich ataxia are reviewed with special regard to cerebellar pathology, pathogenesis, functional changes and possible therapeutic influences, if any. Finally, benefits and limitations of mouse models are discussed.

## Introduction

The cerebellum is a part of the brain that participates in many complex functions. It is involved not only in motor coordination and motor learning but it also plays a role in cognitive and affective functions. Therefore manifestations of cerebellar dysfunction includes motor deficits as well as mental and behavioral abnormalities known in humans as cognitive-affective syndrome [1]. The three main motor signs of cerebellar disorder are ataxia, tremor and increased muscle passivity. These result in many motor problems including ataxic gait and posture, deterioration of goal-directed movements of the extremities, eyeball movement abnormalities, speech disorders, etc. The cerebellum can be damaged due to injuries, ischemia, hemorrhage, tumors, inflammation, intoxication and inherited neurodegenerative conditions. Humans are afflicted with a wide spectrum of hereditary cerebellar degenerations [2], for which, presently, there is no effective causal therapy. The main therapeutic approach is intensive rehabilitation directed toward helping patients improve motor abilities and learning to live with the disease.

Variability of human hereditary cerebellar degenerative disorders is also reflected in animal models of cerebellar ataxias [3]. Mouse models of cerebellar degenerations are either spontaneous mutants or transgenic animals. For some genes there are spontaneous or induced mutations as well as transgenic mouse models. Mouse models of hereditary cerebellar degenerative disorders are used to investigate symptoms, pathogenesis, and cell death mechanisms, as well as to develop and test therapeutic approaches for these diseases. Elucidation of the relationships between functional abnormalities and cerebellar defects could further our understanding of cerebellar function. Since there are complex interconnections between the cerebellum and other areas of the central nervous system and since degeneration, in many cases, is in not restricted to just the cerebellum, a more complete knowledge of the features of individual cerebellar ataxic mice is important for selection of appropriate models for particular studies and for appropriate interpretation of findings. The cerebellar degenerative disorders seen in mice show some similarities with those seen in human patients, although there are also certain differences that limit their research applications. All these fact should be taken into account when choosing and using a mouse model for cerebellar ataxia. The aim of this review is to describe the main types of cerebellar ataxic mice with special regard to cerebellar pathology, pathogenesis, functional changes and possible therapeutic influences, if any, and to discuss their benefits and limitations relative to research applications.

## Review

### Classic cerebellar mutant mice

#### Lurcher mice

Lurcher mice are well studied spontaneous cerebellar mutants. They were first described in 1960 by Phillips [4]. The degeneration is caused by a semi-dominant mutation (*Grid2*^*Lc*^) in the δ2 glutamate receptor (GluRδ2) encoding a gene localized on chromosome 6 [4, 5]. GluRδ2 receptors are expressed at high levels in cerebellar Purkinje cells [6]. The *Grid2*^*Lc*^ mutation is a gain of function mutation, which changes the receptor into a leaky membrane channel that chronically depolarizes the cells [5]. Later, a second Lurcher allele (*Lc*^*J*^), which is phenotypically indistinguishable from *Grid2*^*Lc*^, was found in an inbred strain BALB/cByJ [7].

Homozygous Lurcher mice (*Grid2*^*Lc/Lc*^) die shortly after birth due to the massive loss of mid- and hindbrain neurons during late embryogenesis and the inability to suck for milk after birth [8]. Heterozygous Lurcher mice are viable with normal lifespans and suffer from postnatal degeneration of cerebellar Purkinje, granule, stellate and basket cells and inferior olive neurons [9, 10].

Heterozygous Lurcher Purkinje cell reduction can be detected by about postnatal (P) day 8–10 [9]. The progress of degeneration varies between individual cerebellar lobules [11]. About 95% of Purkinje neurons die between P8 and P25 and virtually all of them have degenerated by P90 [9]. The several hundred surviving Purkinje cells are restricted to the paraflocculus, flocculus and the nodular zone and can be detected as late as P146 [11].

The death of Lurcher mutant Purkinje cells is cell autonomous and it is a primary effect of the mutation [12, 13]. The Purkinje cells show high membrane conductance and a depolarized resting potential due to the presence of a large inward Na^+^ current [5]. This state of permanent cell excitation and over-activation of Na^+^ –K^+^ ATPase probably leads to increased energy demand, decreased intracellular ATP levels [14] and increased mitochondrial cytochrome oxidase activity [15].

Zuo et al. [5] suggested excitotoxic apoptosis as the mechanism of Purkinje cell death in Lurcher mice. Norman et al. [16] observed swelling of axons, chromatin condensation, cell and nuclear membrane blebbing in Lurcher mutant Purkinje neurons, glial cell processes wrapped around dying Purkinje neurons, ready to engulf their remnants. They also observed apoptotic bodies which had already been engulfed by glial cells and the absences of infiltration with leucocytes, which indicated cell death through apoptosis [16]. Deformation of cell shape and nuclei, thickened dendrites, irregular staining of nuclei and cytoplasm and increased numbers of nucleoli in degenerating Lurcher Purkinje cells were also found [17]. Increase in pro-caspase 3 expression, in Lurcher mutant Purkinje cells, may participate in the induction of apoptosis [18]. On the other hand, ultrastructural signs, such as axonal swellings and torpedoes, perinuclear clumps of chromatin and enlarged mitochondria with dilated cristae indicate necrotic cell death [19, 20]. Finally, evidence for autophagic pathways in Lurcher Purkinje cells was also found. Yue et al. [21] reported that dying Lurcher Purkinje cells contain morphological hallmarks of autophagic death in vivo. Later, Wang et al. [22] described accumulation of autophagosomes in axonal dystrophic swellings of Lurcher Purkinje cells. Nishiyama and Yuzaki [14] reevaluated Purkinje cell degeneration in Lurcher mice and designated it as necrosis with autophagic features. Zanjani et al. [23] found that in vitro inhibition of conventional protein kinase C, c-Jun N-terminal kinase, as well as p38, led to enhanced survival of Lurcher mutant Purkinje cells in cerebellar slices suggesting that multiple Purkinje cell death pathways are induced in Lurchers.

Degeneration of granule cells is also fast but not complete. By P60 almost 90% of granule cells are lost [9]. As mentioned above, the number of Golgi, stellate and basket cells are also reduced [10]. Loss of inferior olive neurons becomes apparent by P11 and represents about 70 – 75% of the complete neuronal population [9]. Reduction of granule cells and inferior olive neurons is target-related cell death and can be prevented by surviving Purkinje cells in Lurcher-wild type chimeras [12, 13, 24]. Stellate and basket cells are probably also affected through a target-related cell death mechanism since there is no evidence that the Grid2 receptor is expressed in cerebellar interneurons [10]. Degeneration of the deep cerebellar nuclei is relatively mild in Lurchers [25–27].

Lurcher mice exhibit multiple abnormalities of neural functions. The main and most evident symptom of cerebellar degeneration is ataxia with a wobbly, lurching gait and irregular EMG pattern during walking [28]. Lurchers show poor performance on the treadmill test [29], rotarod test [30–33], static wooden beam [33], unstable platform [34], vertical grid [30, 35], horizontal bar or coat-hanger test [30, 32]. Nevertheless, on some tests, improvement was seen in their motor performance when repeating the task [31, 36, 37]. Lurcher mice show a decline in motor skills [38], motor learning [37] and spatial learning ability [39] with ageing. Lurcher mice also suffer from disorder and lack of adaptive modifications in the oculomotor system [40].

Lurcher mutant mice do poorly on cognitive function tests. Despite this, they show some level of learning ability in the standard Morris water maze test with a hidden platform, although their escape latencies are longer than those of wild type mice [41–43]. Lurchers also have difficulty in guiding themselves, in the water maze, toward a visible goal, which suggests that their deficit in visuomotor coordination contributes to their spatial orientation impairment [41, 44] Lurchers also exhibit deficits in long-term memory [45]. Porras-Garcia et al. [42, 46] described changes in classical conditioning of eyelid responses in Lurcher mice. Lurchers show higher spontaneous activity than control wild type mice [47], however, their exploration behavior on the hole-board test is reduced [35, 47, 48].

While basal levels of both adrenocorticotropic hormone and corticosterone are similar in Lurcher mutants and control mice, mutants show hyper-reactivity of the hypothalamic-pituitary-adrenal axis [49, 50]. Hilber et al. [50] observed that exposure to an anxiogenic situation (elevated plus-maze) increased corticosterone levels more in the mutants than in controls. However, Lurchers showed reduced behavioral indices of anxiety, which suggests that they are rather less inhibited than less anxious [50]. This agrees with observation that Lurchers have lower prepulse inhibition, as described by Porras-Garcia et al. [42]. Nevertheless, stress-provoked high corticosterone levels cause only part of the behavioral disinhibition, since inhibition of corticosterone synthesis produced only modest changes in anxiety-related behaviors in Lurchers [51]. Lurcher females have a high incidence of maternal infanticide, which could be triggered by anxiogenic stimuli linked to their behavioral disinhibition [52].

Furthermore, tests of behavioral flexibility in Lurcher mice and chimeras, between Lurcher heterozygous and wild type individuals, suggested that developmental cerebellar Purkinje cell loss may affect higher level cognitive processes that are commonly deficient in autism spectrum disorders [53]. In Lurcher mice, evoked glutamate release is decreased in the mediodorsal and ventrolateral thalamic nuclei, reticulotegmental nuclei and pedunculopontine nuclei [54]. This is a mechanism by which Purkinje cell loss could disrupt glutamate release in cerebellar efferent pathways that ultimately affects dopamine release in the prefrontal cortex associated with autism spectrum disorders [54].

#### Hotfoot mice

Hotfoot mice have mutation (*Grid2*^*ho*^) in the *δ2* glutamate receptor (GluRδ2) encoding gene on chromosome 6, the same gene, which is affected in Lurcher mice [55]. Several alleles causing the Hot-foot phenotype have been discovered [56, 57]. While the *Grid2*^*Lc*^ (Lurchers) is a gain of function mutation, the *Grid2*^*ho*^ is a loss of function mutation leading to retention of the GluRδ2 in the endoplasmic reticulum and thus its absence on the cell surface [58].

Anatomical alterations are relatively mild; the most obvious being Purkinje cells with ectopic spines devoid of presynaptic innervations [59, 60]. The Hotfoot phenotype is characterized by a flattened body posture, wide base, backing up and jerky movements of the hind limbs [59]. Hotfoot mice do poorly on the rotating grid, wooden beam, coat hanger and rotarod tests, however, they showed evidence of learning in the rotating grid and coat hanger tests, but not on the wooden beam and rotarod [31, 36, 61]. They did not alternate above chance in the T-maze and failed in the Z-maze test [62].

#### Purkinje cell degeneration mice

Purkinje cell degeneration (pcd) mice are one of the most frequently used spontaneous cerebellar mutants. The heredity of the disorder is autosomal recessive with full penetrance [63]. Pcd mice are homozygous for the *Agtpbp1*^*pcd/J*^ mutations in the gene encoding cytosolic ATP/GTP binding protein 1 (synonyms: cytosolic carboxypeptidase-like protein, CCP1, Nna1) located on chromosome 13 [64]. Several spontaneous mutant alleles (*Agtpbp1*^*pcd-1J*^, *Agtpbp1*^*pcd-2J*^, *Agtpbp1*^*pcd-3J*^, *Agtpbp1*^*pcd-5J*^, *Agtpbp1*^*pcd-7J*^) and induced mutations (*Agtpbp1*^*pcd-4J*^, *Agtpbp1*^*pcd-6J*^) of the gene with similar phenotypes have been discovered or generated, respectively. CCP1 is a metallopeptidase that plays a role in peptide turnover. In normal mice it is intensively expressed in cerebellar Purkinje cells, olfactory bulb mitral cells and retinal photoreceptors [64]. Therefore, pcd mice postnatally lose virtually all Purkinje cells and suffer from slow, progressive degeneration of the retina and olfactory bulb mitral cells.

The primary cerebellar pathology in pcd mutants is the loss of Purkinje cells. On P15 the number of Purkinje cells is still within normal range, however, they have already started to show structural abnormalities [65]. Purkinje cell loss starts at P20 and by P25 it has already become massive [66]. By P28 it is nearly complete in most parts of the cerebellum except for the most ventral areas, where numerous Purkinje neurons still remain [63]. Only a few Purkinje cells remain in the nodulus after 7 weeks [63].

Kyuhou et al. [67] suggested that the death of Purkinje cells is apoptotic via the activation of caspase 3. Chakrabarti et al. [68] later described increased autophagy in Purkinje cells. Berezniuk and Fricker [69] suggested that the lack of CCP1 leads to decreased levels of cellular amino acids, which then induce increased autophagy. This could be a protective response to cellular amino acid starvation. They also reported increased autophagy in brain structures, which do not undergo degeneration in pcd mice [70]. CCP1 has also been shown to catalyze shortening of glutamate side chains on polyglutamylated brain tubulin [71, 72]. Hyperglutamylation resulting from the loss of the function of the enzyme in pcd mice has been suggested to be the primary cause of the degeneration [71].

The degeneration of cerebellar granule cells is secondary to the loss of Purkinje neurons and is exponentially progressive [73, 74]. The moderate reduction in the size of deep cerebellar nuclei in older mutants is probably due to loss of synaptic input from Purkinje cells [75]. Inferior olivary neurons start to disappear between P17 and P23 and by P300 the reduction has reached 49% [73]. The degeneration is probably due to the loss of their postsynaptic targets, i.e. Purkinje cells [73]. Degeneration of Purkinje cells is followed by activation of microglia and astrocytes in the cerebellum and apoptotic death of cerebellar oligodendrocytes [66, 67].

Olfactory bulb fiber degeneration starts at 13 weeks and at 38 weeks it is massive. Older animals lose mitral cells in the olfactory bulb [63]. Degeneration of the thalamus has also been observed in the brain of pcd mice. Rapid degeneration between P50 and P60 affects the majority of neurons in the central division of the mediodorsal nucleus, ventral medial geniculate, posterior ventromedial and submedial nuclei and those parts of the ventrolateral and posteromedial nuclei surrounding the medial division of the ventrobasal complex [76]. Subtle changes were also found in other thalamic nuclei [76].

Retinal degeneration is manifested by pycnotic nuclei of photoreceptors at 3 weeks in homozygous pcd mice; 50% of the photoreceptors are gone by the fifth week and the remaining photoreceptors are gone within one year [77–79]. The progress of degeneration is mirrored by decline in retinal function [80].

Pcd mice have reduced bodyweight [63, 81] and poor overall health. Pcd males are sterile due to low concentrations, low motility and abnormally formed sperm [63]. Females are not sterile, however their fertility is reduced and they have difficulties rearing pups.

The major neurological symptom in pcd mice is cerebellar ataxia that becomes apparent around P21 [63, 81]. Pcd mice perform poorly on the coat-hanger and rotarod tests but perform well on the rectangular as well as round stationary beam [82]. Goodlett et al. [81] reported that pcd mice were unable to navigate to a hidden goal in the Morris water maze but had good performances on visual guidance tasks. This finding shows that pcd mice are able to navigate to visible goals, but spatial navigation based on multiple distal cues is severely impaired [81]. Pcd mice show deterioration of delayed eye-blink classical conditioning [83] but not the trace one [84].

Although the *Agtpbp1*^*pcd*/*J*^ mutation is known to be recessive, it has mild effect on Purkinje cells also in heterozygous individuals. At P150 there are no differences in Purkinje cell number between wild type and heterozygous pcd mice, however, by P300 there is 20% reduction in heterozygotes [85]. Doulazmi et al. [86] reported a similar (18%) reduction at 17 months, in heterozygous pcd mice. This mild degeneration has been shown to promote fusion of surviving Purkinje cells with grafted bone marrow-derived cells [85].

#### Nervous mice

Nervous mice are autosomal recessive mutants suffering from a severe degeneration of Purkinje cells. The nervous mutation (*nr*) is located on chromosome 8 [87, 88]. The Nervous phenotype shows incomplete penetrance and the existence of a nervous modifier locus on mouse chromosome 5 is supposed [88].

On P9 abnormal rounded mitochondria appear in some Purkinje cells and by P15 abnormal mitochondria are present in all Purkinje cells [89]. Later, most of the cells display progressive degenerative changes in the rough endoplasmic reticulum, Golgi complex and polysomes. As late as P23 the number of the Purkinje cells is still almost normal, however, rapid degeneration follows and by P50 80% of Purkinje cells in the vermis, 88% along the vermis-hemisphere junction and 97% in the hemispheres are gone [89]. Although every nervous Purkinje cell has spherical mitochondria, 10% of the Purkinje cells do not degenerate and their mitochondria reacquire normal shape [89]. Purkinje cell necrosis is mediated by tissue plasminogen activator, levels of which are elevated in the cerebellum of homozygous nervous mutant mice [90, 91]. As a consequence of Purkinje cell loss, one third of the inferior olive neurons undergo retrograde degeneration [92]. Berrebi and Mugnaini [93] described alterations in the dorsal cochlear nuclei of Nervous mice that were similar to those seen in Purkinje cells and reported that although most of the cartwheel cells of the nuclei survive, they show abnormal mitochondria throughout adulthood.

Nervous mutant mice are characterized by their hyperactivity and ataxia [94]. They show impairment on the stationary beam, coat-hanger, and rotarod tests of motor coordination, impairment on the submerged platform, but not the visible platform, Morris water maze task and with higher levels of motor activity in an automated chamber but normal activity on the open field test [94]. The phenotype of nervous mice also includes lower body weight than wild type mice with the same background [94].

The Nervous mutation also leads to retinal degeneration [95]. Photoreceptors degenerate rapidly between P13 and P19, and then degeneration slows [96]. By 7.5 months the outer nuclear layer of the retina consists only of a single row of nuclei, most of which are lost later so that in 17 months old mice only a few photoreceptors remain [96].

#### Staggerer mice

The Staggerer is an autosomal recessive mutation (*Rora*^*sg*^) in the gene encoding the retinoid-related orphan receptor alpha on chromosome 9 [97]. In their first description Staggerer mice were characterized by a staggering gait, tremor hypotonia, small body size and a cerebellar cortex having few granule cells and unaligned Purkinje cells [98].

Development of Purkinje cell spines is delayed in Staggerers. The spines appear later on both the cell soma and dendrites and their density is lower [99]. Cytological appearance and degeneration of Purkinje cells is regionally variable and by P30 between 60% and 90% are gone [100]. Surviving Purkinje cell somata and dendrites are smaller, ectopic and the dendrites are not confined to the sagittal plane [101, 102]. The regional variability of Purkinje cells along the mediolateral axis seems to correspond with variability in calbindin expression [103]. The external granular layer is thinner and the number of postmitotic granule cells is reduced by P21 and almost all granule cells have degenerated by P28 [101]. Staggerer mice lack synapses between Purkinje cells and parallel fibers before granule cells disappear. On the other hand, regression of multiple innervations of Purkinje cells, by climbing fibers, fails and so several of these fibers synapse with each Purkinje cell instead of a single one as in normal mice [104, 105].

A study using Staggerer-wild type chimeras revealed that in the Staggerer-genotype medium-to-large neurons in the cerebellum expressed all the defects present in homozygous Staggerer mice, while genotypically wild type Purkinje cells had a normal appearance, suggesting that Purkinje cell defects are cell-intrinsic [102, 106]. On the other hand, Staggerer granule cells were rescued in the chimeras and their number had a linear relationship with the number of Purkinje cells, thus granule cell death is an indirect consequence of the mutation [24, 107, 108].

Deep cerebellar nuclei, despite being reduced in volume by 30% have normal numbers of neurons [109, 110]. There is also marked disorganization and loss of almost 60% of the cells within the inferior olivary complex due to target-related cell death, which is secondary to the degeneration of Purkinje cells [111]. Despite the *Rora*^*sg*^ mutation being described as a recessive mutation, heterozygous mutants, at 12 months, have undergone degeneration of 35% of Purkinje cells, 35% of granule cells and 40% of inferior olive neurons [112]. The *Rora*^*sg*^ mutation also influences neuronal differentiation and development in the hippocampal dentate gyrus as shown by a lower expression of doublecortin and NeuN [113].

Staggerer mice display an enhanced endocrine response to novelty stress, which lacks the diurnal shift in corticosterone nonstress levels [114]. Staggerers have multiple behavioral and motor abnormalities. They have an unsteady gait, shorter fall latencies on the wooden beam, grid [115] and rotarod tests [116]. On the rotating grid, wooden beam and coat hanger tests, Staggerer mice had worse performances than wild type controls and they were not able to improve when the tasks were repeated for 7 days [31]. They performed poorly on the radial arm maze and active avoidance tasks [117]. Staggerer mice had fewer hole visits in the hole board test [115]; however, they explored novel objects in a familiar environment for longer times [118] and showed a tendency to return to the place in a maze that they had most recently visited, probably due to abnormal novelty reactions [119]. Since their cerebellum is already abnormal at birth, newborn Staggerers are less efficient in specific motor tasks, have lower body weight and differ from wild type mice in ultrasound production [120].

#### Weaver mouse

Weaver mice are semi-dominant mutants carrying the missense *Grik*^*Wv*^ mutation of the gene encoding a G-protein coupled with inward rectifying potassium channel and located on chromosome 2 [121]. The mutation results in a disorganized cerebellar structure [122, 123] and also affects several extra-cerebellar brain areas.

By the time the pups are born, cell death is detectable in the external granular layer of both homozygous and heterozygous Weaver mice [124]. After birth, abnormalities develop dramatically when the granule cells begin to migrate, so that by P10 developmental defects are markedly present [123]. At this age, the cerebellum of heterozygous Weaver mice is reduced by 5-10%, the granular layer is thinner, while the external granular layer is disarranged and wider than in wild type mice and Purkinje cells are less strictly aligned [123]. The molecular layer is narrower, but shows a greater density of cell bodies because it contains young granule cells [123]. Those cells having a typical soma are properly aligned with normal Bergmann glial fibers, many of the anomalous cells lie contiguous with abnormal glial processes and cells that have lost contact with glial cells are usually seen in various stages of degeneration [123]. In homozygous Weaver mice, cerebellar abnormalities are much more pronounced. The external granular layer is indistinct, granule cells form irregular vertical stacks and many of them have degenerated and Purkinje cells are arranged in several rows and have randomly oriented dendritic trees [123, 125]. As in Staggerer mice, Weaver mice Purkinje cells receive multiple innervations from climbing fibers [126]. Later in life cerebellar cytoarchitecture improves somewhat in heterozygous Weaver mice, while most of the homozygotes die around weaning time [123].

Contrary to Lurcher, pcd and Nervous mice, Purkinje cell loss is mild in Weavers. Heterozygotes have 86% and homozygotes 72% of the normal number of Purkinje cells [127]. The reduction in the number of Purkinje neurons, as well as granule cells, is more severe in the medial parts of the cerebellum than in lateral areas of the hemispheres [128]. Homozygous Weaver mice suffer from a 20-25% decrease in the number of deep cerebellar nuclei neurons [129]. The inferior olive of both homozygous and heterozygous weaver mice is normal, olivocerebellar projections have a normal topographic organization [127] and most cerebellar afferent pathways appear to be unchanged [130, 131]. On the other hand, Ozaki et al. [132] described degeneration of pontine nuclei neurons and retraction of mossy fibers from the cerebellar cortex.

The fact, that degenerating granule cells had abnormal or no contact with Bergmann glia led to the suggestion that the degeneration and reduced rate of migration of granule cells is secondary to a disorder of Bergmann glial cells [123, 133]. Later studies on chimeric mice revealed that while ectopic Purkinje cells were of both Weaver and non-Weaver origin, all ectopic granule cells were from the weaver component of the chimera, thus the abnormality seen in granule cells is intrinsic and a direct effect of the *Grik*^*Wv*^ mutation [134]. Furthermore, degeneration affects Weaver Purkinje cells but not non-Weaver Purkinje cells, indicating that while the disorganization of Purkinje cells is an indirect effect, their degeneration is a direct result of the *Grik*^*Wv*^ mutation [135].

Despite the absence of parallel fibers, Weaver Purkinje cells have normal initial development of both somatic and dendritic spines [136]. They grossly resemble normal Purkinje cells electrophysiologically, when the only qualitative difference is in response to acetylcholine, which increases their firing rate instead of having its usual inhibitory effect [137].

Besides the cerebellum, changes in several other areas of the CNS have been found in Weaver mutants. Cell loss has been described in the pars compacta of the substantia nigra, ventral tegmental area and the retrorubral nucleus [138–140]. In the hippocampus of homozygous Weaver mice, a thicker pyramidal cell layer in the CA3 area with cell-free spaces, ectopic clusters of pyramidal cells, on occasions subdivision of the pyramidal cell layer into 2–3 layers and disorganized mossy fiber projections have been described [141]. The retinas of adult Weaver mice contain more dopaminergic cells, some of which have an abnormal appearance and location [142]. Retinopetal tyrosine hydroxylase-immunoreactive fibers are also dramatically increased in number [143].

Weaver mutants do poorly on the spatial navigation in the water maze test [144]. In the forced swimming task, they did not acquire the immobility response, which was seen wild type mice [145]. Weaver mice had less exploratory activity on the hole board test and shorter fall latencies on the wooden beam and grid tests [146]. They also showed learning deficits [147]. Like some other cerebellar mutations, the *Grik*^*Wv*^ mutation leads to male sterility due to the death of germ cells in the testes of homozygous males [148].

Motor performance of Weaver mutants has been shown to improve after removal of the cerebellum, which eliminated the faulty output of the surviving Purkinje cells [149]. This suggests that dysfunctional Purkinje cells can be worse than no Purkinje cells at all. Granule cells death has been identified as being apoptotic [150, 151]. In vitro studies showed that Weaver granule cells can be rescued by pharmacological blockade of sodium influx [152], with the calcium channel blocker verapamil, with high concentrations of the glutamate receptor antagonist MK-801, using antibodies against the B2 chain of laminin [153] and by protease inhibitors [154]. Nevertheless, removal of tissue plasminogen activator, a serine protease, which is elevated in the cerebellum of Weaver mice, did not protect against the degeneration of granule cells [155]. Overexpression of insulin-like growth factor-I was found to protect Weaver granule cells *in vivo*, improve muscle strength and alleviate ataxia [156].

#### Reeler mouse

Reeler mice [157] have an autosomal recessive *Reln*^*rl*^ mutation on chromosome 5 [158]. The gene encodes the extracellular matrix protein reelin, which is important for neural cell migration [159]. The mutation leads to disordered neuron migration during CNS development resulting in architectonic disorganization and ectopic cell localization in the cerebellum [160, 161], hippocampus [162, 163], neocortex [164], inferior olive [165], olfactory bulb [166], cochlear nucleus [167], superior colliculus [168] and substantia nigra [169]. The characteristic feature of the Reeler brain cortex is an inversion of the layers and cells [170]. Nevertheless, most of the structures are appropriately interconnected [171–175].

The cerebellum of homozygous Reeler mice is reduced in size [176]. Since embryonic day 17, foliation of the cerebellum becomes deficient [177]. A study of the histological structure of the Reeler cerebellum revealed deep alteration of the cerebellar cortex architecture consisting of out of place Purkinje cells in various locations, lower density of granule cells, lower density of synaptic contacts between Purkinje cells and parallel, climbing and mossy fibers and basket cells [176, 178, 179]. Purkinje cells are reduced to slightly less than half the normal number and only 5% of the surviving ones are positioned normally, i.e. located between the molecular and granular layers, while 10% are located within the granular layer and the majority are in characteristic subcortical cellular masses [180]. Some granule cells are retained in the molecular layer. Their axons have an abnormal course and in some cases have no T-like bifurcation [181]. The distribution of fibers within the white matter and their arborization are changed as a result of the cerebellar architecture disorganization [182]. Despite all the above mentioned disarrangement, the specificity of most of the connections is preserved [176]. Some abnormalities, however, have been discovered: (1) deep Purkinje cells receiving several climbing fibers, (2) ectopic somato-dendritic or dendro-dendritic synapses between granule and Purkinje cells and (3) synapses between mossy fibers and Purkinje cell spines appearing in the granular layer and central mass [176, 183]. Despite this, intracellular recordings showed that Reeler Purkinje cells have normal sodium- and calcium-dependent spikes [184]. The homozygous Reeler inferior olivary complex is reduced in size by 22.6% [127].

During brain development, the upward migration of young neurons is terminated in the depths of the neocortex; additionally, abnormally extensive contacts between glial fibers and somata of post-migratory cells appear to be sustained in Reeler mutants suggesting that abnormal adhesions between post-migratory cells and radial glial fibers obstruct neuronal migration [185]. Within the cerebellum, the role of dysgenesis of radial glia in obstructed migration of Purkinje cells has been shown by Yuasa et al. [186]. Goffinet et al. [179] suggested that the primary defect in the Reeler cerebellum is malposition of Purkinje cells and that the mutation affects the terminal phase of migration of these cells in the cerebellum. However, findings of normal cerebellums in normal mouse-Reeler chimera suggest that the disturbance of neuronal migration in Reelers is attributable to abnormal cell-to-cell interactions between young neurons and the radial glia [187] and is not determined cell-autonomously [188].

Reeler mice have lower neurogenesis rates and increased susceptibility to ischemic brain injury [189] and to epileptic seizures [190]. In humans, autosomal recessive lissencephaly is associated with mutations in the reelin encoding gene; and as with Reeler mice, neuronal migration is impaired and abnormalities in the cerebellum develop [191].

Reeler mice have an interesting behavioral phenotype. Homozygous Reeler mice performed poorly on the active avoidance task and on the radial arm maze task; however, they were able to improve to a level similar to wild-type controls after training [117]. Reeler mice also performed poorly on the spontaneous alternation task in the T-maze, water maze, stationary beam, coat-hanger and rotarod tests [192].

Various behavioral abnormalities have also been reported in heterozygous Reeler mice. Heterozygous Reeler mice have a deficit in learning olfactory discrimination [193]. Young heterozygous Reeler mice showed significantly lower levels of anxiety- and risk-assessment-related behaviors in the elevated plus-maze, whereas adult mice exhibited elevated levels of motor impulsivity and altered pain threshold [194]. Heterozygous mice also exhibited complex changes in startle reactivity and sensorimotor gating [195]. The vocal repertoire of neonatal Reeler mice is characterized by preferential use of a specific two-component call [196]. In heterozygous mice the number of calls is increased and the ontogenetic peak in the frequency of calls is delayed compared to wild type mice, while in homozygous Reeler mice the peak is absent [196]. On the other hand, a detailed behavioral study by Podhorna and Didriksen [197] did not find any differences between heterozygous Reelers and wild type mice. Qiu et al. [198] also reported similar performance in heterozygous Reelers and wild type mice, but found a reduction in contextual-fear-conditioned-learning and impaired hippocampal long-term potentiation in heterozygotes.

Some neurobehavioral abnormalities reported in Reeler mice are thought to parallel some features found in human psychiatric disorders and heterozygous Reeler mice have even been suggested as a model of schizophrenia [199, 200]. On the other hand, Krueger et al. [201] do not consider heterozygous Reeler mice to be an appropriate model for schizophrenia but rather of general learning deficits associated with many psychiatric disorders.

Several experimental therapies have been developed for heterozygous Reeler mice. Cysteamine treatment was found to improve prepulse inhibition and Y-maze performance [202]. Nicotine treatment elevated reelin in the brain to wild type levels and normalized hyperactivity [203]. Reelin supplementation was also found to enhance associative learning ability and prepulse inhibition [204].

#### Scrambler mouse

The spontaneous autosomal recessive scrambler mutation (*Dab1*^*scm-3J*^) in the disabled-1 (DAB1) gene on chromosome 4 was described by Sweet et al. [205]. The cerebellum of one month old homozygous Scrambler mice is hypoplastic and devoid of folia [205]. Granule cells numbers are reduced by 80%, Purkinje cells numbers are also reduced and only 5% are normally located [206]. Despite Purkinje cell ectopia, topographic afferents remain conserved [207].

Homozygous Scrambler mice suffer from an unstable ataxic gait and whole body tremor [205] that distinguishes them from controls by as early as P8 [208]. Scrambler mice performed poorly on the rotarod, stationary beam and coat-hanger tests [209], but were able to improve their performance on the vertical grid [210] and were more active in the open field [209]. In the alternation test, Scrambler mice did not alternate above chance levels and they were impaired in both the hidden and visible platform Morris water maze tasks [211].

The Scrambler phenotype is similar to Reeler mice [205, 212] despite normal levels of reelin in Scrambler mice [206]. This is because function of extracellular reelin, which is affected in Reeler mice depends on the intracellular protein disabled-1 [206, 213].

## Mouse models of human hereditary cerebellar ataxias, genetically engineered and new cerebellar mutants

New sources of cerebellar mutant mice include transgene technology, induced and targeted mutations and mutagenesis enhanced with mutagen substances [214, 215]. The latter approach is capable of providing large numbers of random mutations carriers that can be identified according to the abnormal phenotype. Many mouse models of cerebellar disorders, namely those genetically engineered, are related to particular human disease.

### SCA1

Spinocerebellar ataxia type 1 (SCA1) is one of the autosomal dominant hereditary ataxias. It is caused by an enlarged region of CAG trinucleotides repeat in the ataxin-1 gene which results in a poly-glutamine tract (polyQ) expansion in the ataxin-1 protein. Normal length varies between 6 and 35 repeats, while in SCA1 patients it is 39–83 (for review see [216]).

Burright et al. [217] generated transgenic SCA1 mice carrying the allele with 82 CAG repeats under the control of the murine Pcp2 promoter, which is supposed to be capable of directing transgene expression specifically to Purkinje cells. Five lines of the mice showed transgene expression levels 10-100-fold greater than levels of endogenous mouse ataxin-1 mRNA [217]. Morphologic changes had varying intensities depending on the transgenic mouse line and on the presence of heterozygous or homozygous combinations of the transgene [217]. Morphologic changes consisted of a marked loss of Purkinje cells, abnormal Purkinje cell dendritic trees, Bergmann glia proliferation, shrinkage and gliosis of the molecular layer and the presence of ectopic Purkinje cells in the molecular and granular layers [217]. Progressive ataxia appeared earliest (at the age of 12 weeks) in the line with the highest transgene expression; however, among other lines ataxia onset and severity were not strictly correlated with transgene mRNA [217]. Clark et al. [218] described the same SCA1 mice shorter strides at 12 weeks and dramatic gait pattern abnormalities at 1 year, and diminished performance on the rotarod test obvious by 5 weeks.

Transgenic mice helped in the discovery of some possible components of the pathogenesis of SCA1. Klement et al. [219] demonstrated that while nuclear localisation of pathological ataxin-1 is necessary, its nuclear aggregation is not required to initiate pathogenesis in transgenic mice. Cummings et al. [220] suggested that impaired proteasomal degradation of mutant ataxin-1 may contribute to SCA1 pathogenesis in transgenic mice. Expanded ataxin-1 has been found to mediate downregulation of genes involved in signal transduction and calcium homeostasis in SCA1 mice prior to manifestation of the pathology [221].

### SCA2

Transgenic mice carrying the human ataxin-2 gene, with an enlarged CAG repeat sequence, are used as a model of human spinocerebellar ataxia type 2 (SCA2). Normal ataxin-2 usually contains 22 or 23 glutamines. SCA2 patients have 32–77 repeats (for review see [216]). SCA2 transgenic mice were generated in several lines with different lengths of the CAG repeat segment and varying in severity of disease manifestation.

Transgenic SCA2 mice with 58 CAG repetitions in the ataxin-2 gene (Q58) under control of the Pcp2 promoter were generated in 3 founder lines (Q58-5B, Q58-11, Q58-19) on the B6D2 hybrid background by Huynh et al. [222]. Purkinje cell numbers were reduced by about 50% in mice 24–27 weeks old. Mice from the Q58-19 line showed altered stride length by the 8 weeks and by 16 weeks stride length was reduced in all 3 lines [222]. At 6 weeks, transgenic mice did not differ on the rotarod test from wild type controls, homozygous and heterozygous mice from the Q58-11 line were performing poorly by 16 or 26 weeks respectively. Mice from the Q58-5B line had performances similar to those from the Q58-11 line [222].

Aguiar et al. [223] described a similar pathology in mice (B6D2 hybrid background) carrying the SCA2 transgene with 75 CAG repeats (Q75) under SCA2 self-promoter regulation, including earlier onset of symptoms in homozygous compared to heterozygous individuals. Dispersion of ataxic symptom onset time was higher in heterozygotes [223].

Transgenic SCA2 mice (B6D2 hybrid background) expressing Q127 ataxin-2 under control of Pcp2 promoter have been shown to have a reduction in the expression of genes specific for Purkinje cells (such as Calb1, Pcp2, Grid2), to have a decrease in Purkinje cell firing frequency first at 6 weeks and progressive motor performance deterioration identified, using an accelerating rotarod test, began at 8 weeks [224]. Motor decline, electrophysiological abnormalities as well as gene expression changes preceded the decrease in Purkinje cell number, which occurred several weeks later [224].

It seems, that the manifestation was more sever in mouse lines with longer polyQ tracts and this phenomenon is in agreement with the inverse correlation observed between CAG repeat length and the age of onset in human patients [225]. Moreover, experiments with transgenic SCA2 mice clearly show that the homozygous combination of the pathologic allele, which is unusual in humans, leads to an earlier onset of symptoms.

Studies on transgenic SCA2 mice have provided important information about certain aspects regarding the pathogenesis of the disease. Huynh et al. [222] showed that nuclear localization and inclusion body formation of ataxin-2 are not necessary for disease development. Liu et al. [226] demonstrated that (1) glutamate induced more pronounced cell death in the Q58 Purkinje cell culture than in wild type cells, (2) glutamate-induced cell death was attenuated by dantrolene, a calcium ion stabilizer, and (3) long-term treatment of SCA2-58Q mice with dantrolene alleviated motor deficits and Purkinje cell loss. These findings suggested that disturbed calcium signaling may play a role in the pathogenesis of SCA2 [226].

### SCA3

Spinocerebellar ataxia type 3 (SCA3) is also known as Machado-Joseph disease. It is a late-onset degenerative disease caused by a CAG repeat expansion in the gene encoding the ataxin-3 protein. Within normal alleles, repeat length varies between 12 and 44 CAG trinucleotides. In SCA3 patients, the length is 52–86 (for review see [216]).

Transgenic mice with alleles containing polyglutamine tracts of 64, 67, 72, 76 and 84 repeats have been generated by Cemal et al. [227]. These transgenic mice suffer from a loss of neurons in the pontine nuclei, increased numbers of reactive astrocytes in the dentate nucleus, cerebellar white matter and moderate degeneration of Purkinje cells, which showed dependence on the transgene copy number [227]. The mice had a wide gait during grid climbing, lowered pelvises, tremor, lower activity, clasping, and slow weight gain [227]. They also had features suggesting peripheral neuropathy characterized by demyelination and degeneration of the dorsal root ganglia [227].

Transgenic mice expressing ataxin-3 with an expanded polyglutamine tract with 79 repeats suffer from progressive ataxia with onset at 5–6 months, followed by progression, despite no prominent neuronal loss in the cerebellum, even as late as 10–11 months [228]. This suggests that neuronal dysfunction and down-regulation of cerebellar expressions of proteins involved in synaptic transmission, signal transduction or regulation of neuronal survival and differentiation, rather than Purkinje cell loss, could be responsible for the decline in cerebellar function [228, 229]. Studies on the SCA3 transgenic mouse model have suggested that Purkinje neuron dysfunction associated with altered voltage-activated potassium channels [230], calcium-dependent calpain-type proteases [231], and disruption of dendritic development and metabotropic glutamate receptor signaling in Purkinje cells by mutant ataxin-3 [232] may also play role in the pathogenesis of SCA3. Soluble extended ataxin-3 has shown a tendency for decrease during disease progression in the cerebellum and to inversely correlate with aggregate formation and phenotypic aggravation in SCA3 mice [233].

Boy et al. [234] and Nobrega et al. [235] showed, that symptoms can be alleviated with pathological ataxin-3 expression silencing in diseased transgenic SCA3 mice. Halting the expression of ataxin-3 is seen as a hopeful therapeutic approach since the technique cleared nuclear accumulation of the abnormal protein in SCA3 mice [236]. Stimulation of proteasome activity by Rho-kinase alleviated the neurological phenotype in SCA3 mice since it may promote mutant ataxin-3 degradation [237].

### SCA6

Spinocerebellar ataxia type 6 (SCA6) is due to a CAG repeat expansion in the CACNA1A gene encoding the alpha 1A-voltage-dependent calcium channel (CaV2.1) [238]. Normal alleles have 4–16 repeats, while alleles causing disease contain 20–33 repeats (for review see [216]). Different mutations in the same locus appear in ataxic mouse mutants Tottering [239, 240], Leaner [241] and Rocker [242].

Mice expressing CaV2.1, with the 84 polyglutamine tract, developed progressive motor impairment and aggregation of the mutant protein [243]. Homozygous mice exhibited hypoactivity at 17 months and poor performances on the accelerating rotarod [243]. Heterozygotes were indistinguishable from wild type controls based on visual inspection up to 20 months, however, at 19 months they were underperforming on rotarod test [243]. No neuronal loss, no changes in Purkinje cell morphology and no alterations in calbindin immunoreactivity were found in these mice, even as late as 20 months, when motor deficits were already apparent [243].

Studies on SCA6 mice with 28 or 84 polyglutamine repeats have shown that voltage dependence of activation and inactivation, and current density measured in Purkinje cells were not influenced by the polyglutamine tract length, which suggests that alteration of CaV2.1 channel properties does not play a role and that the pathogenesis of SCA6 may be linked to accumulation of mutant channels [243, 244]. The decreased whole cell current density could be attributed to a decrease in CaV2.1 channel numbers [243]. In Tottering mice, a mutation in the CACNA1A gene leads to irregular simple spike activities in Purkinje cells without any change in other activity parameters and this abnormality has been suggested as being sufficient to produce behavioral abnormalities [245].

### SCA7

Spinocerebellar ataxia type 7 (SCA7) is a neurodegenerative disease caused by expansion of a CAG repeat within the gene encoding ataxin-7. The normal range is 7–19 repeats. Pathological alleles contain from 37 to more than 400 CAG triplets. Among SCAs, it is the only one characterized by severe ataxia and at the same time by visual loss due to pigmentary macular degeneration (for review see [216]).

Nuclear inclusions of mutant ataxin-7, motor coordination and vision impairment have been reported in mice overexpressing full-length mutant ataxin-7 with 90 glutamines (Q90) in Purkinje cells and photoreceptors [246]. Yoo et al. [247] generated an authentic mouse model of SCA7 by targeted insertion of 266 CAG repeats into the mouse SCA7 locus. The mice displayed an infantile form of SCA7 that is manifested by ataxia, visual deficit, impaired short-term synaptic potentiation and premature death [247]. In transgenic mice, expression of the Q92 ataxin-7 in all CNS neurons, except for Purkinje cells, led to severe Purkinje cell degeneration, development of gait ataxia, and formation of truncated ataxin-7 nuclear aggregates that correlated with disease phenotype onset and even to premature death [248]. These findings suggest that the degeneration of Purkinje cells is non-cell-autonomous in SCA7 and that lack of proteolytic cleavage may be one of the SCA7 pathogenetic mechanisms [248]. The next feature of SCA7 pathogenesis may be changes in gene expression. Mice expressing Q52 ataxin-7 and exhibiting motor dysfunction since the age of 7 months had down-regulated mRNA expression of proteins involved in glutamatergic transmission, signal transduction, myelin formation, axon transport, deubiquitination, neuronal differentiation and glial function [249]. Contrary to mice expressing Q92 ataxin-7, mice expressing Q52 did not lose significant numbers of Purkinje cells, despite ataxic symptoms [249].

Experimental therapeutic approaches have been successfully tested in SCA7 transgenic mice. Suppression of mutant gene expression by 50%, started one month after the ataxia onset, was effective at halting or reversing motor symptoms, reducing mutant ataxin-7 aggregation in Purkinje cells and preventing synaptic loss between climbing fibers and Purkinje cells [250]. As with SCA3 mice, augmenting proteasome activity promoted mutant ataxin-7 degradation [237]. It has also been shown that interferon beta was able to induce clearance of ataxin-7 and improved motor function in Q266 SCA7 mice [251].

### SCA23

SCA23 is a rare disease caused by mutations in the gene encoding prodynorphin (for review see [216]). Prodynorphin knock-out mice are more sensitive to noxious stimuli but have normal responses to non-noxious stimuli [252]; additionally, mutant dynorphin proteins have enhanced non-opioid excitatory activities which may underlie development of SCA23 [253]. Nevertheless, these mice have not been tested yet for phenotypic similarity to human SCA23.

### DRPLA

Dentatorubral-pallidoluysian atrophy (DRPLA) is an autosomal dominant neurologic disorder. It manifests as variable combinations of cerebellar ataxia, dementia, epilepsy and choreoathetosis. It is caused by an unstable expansion of CAG repeat in the gene encoding atrophin-1 on chromosome 12 [254]. The age at symptom onset is highly variable and correlates with the length of the polyglutamine tract [254].

Transgenic mice with 78 CAG repeats in the DRPLA gene showed intergenerational instability of the CAG repeat tract, which is typical for human DRPLA [255]. Mice with 76 CAG triplets also revealed instability but no obvious neurological abnormalities [256, 257]. Mice having 129 repeats revealed a marked neurological phenotype, age dependent dysfunction of the globus pallidus and cerebellum, shrinkage of the distal dendrites of Purkinje cells, intranuclear accumulation of mutant protein and progressive brain atrophy, but no neuronal loss [256, 257]. Changes in gene expression in the cerebellum and cerebrum, due to intranuclear accumulation of mutant DRPLA protein have been shown [258]. Increase in the severity of motor deficits that parallels the length of the expanded CAG repeats and with age, has been observed in mice carrying full-length mutant human DRPLA gene with 76, 96, 113 or 129 CAG repeats.

### Niemann-pick disease model

Niemann-Pick disease is an autosomal recessive inherited storage disease with types A, B and C. Its manifestation is heterogeneous and consists of extraneural and CNS (in type A and C) affections, with depletion of Purkinje cells being seen in type C. Mutations causing the analogous disease in mice have been described and transgenic mouse models for Niemann-Pick disease type C have been created [259–261]. Poor performances on motor tests and also on the water maze task with the hidden escape platform have been reported in these mice [262, 263]. Cerebellar affection, however, is only one of multiple pathologies affecting these mice. Therefore this model is not suitable for studying cerebellar functions. Nevertheless, it has been successfully used several times to investigate the pathogenesis of Niemann-Pick type C disease [264, 265] and to assess pharmacological [266] and transplantation [267–270] methods that could potentially be used to treat those afflicted with Niemann-Pick type C disease.

### Friedreich ataxia

Friedreich ataxia (FRDA) is a human autosomal recessive disease caused by a GAA triplet repeat expansion in the gene encoding the frataxin, a mitochondrial protein involved in iron metabolism (for review see [271]). Several transgenic mouse models have been generated. Al-Mahdavi et al. [272] reported vacuoles within dorsal root ganglia neurons, axons demyelination, coordination deficits and decreased locomotor activity. The next mouse model with a frataxin transgene containing 230 GAA repeats and reduced expression of wild type frataxin did not develop motor coordination impairment and iron metabolism anomalies [273]. Several other transgenic mice carrying pathological frataxin gene alleles have yet to be tested for Friedreich ataxia phenotype.

### Robo3-deficient mouse

Robo3 (known also as Rig-1 or HGPPS), a member of the roundabout family of transmembrane receptors, has been shown to be important for neurons and axons to cross the midline in mice [274, 275]. Robo proteins also regulate midline crossing of precerebellar neurons and cerebellofugal axons [275–277]. In humans, Robo3 is related to a syndrome involving horizontal gaze palsy with progressive scoliosis (HGPPS) [278, 279]. Robo3 deficient mice are not a model of cerebellar degeneration but rather a developmental disorder of the laterality of projections from axons including those connecting the cerebellum with other CNS structures. Renier et al. [280] generated Ptf1a::cre;Robo3^lox/lox^ mice lacking the interolivary commissure. While in normal mice inferior olive neurons project contralaterally to the cerebellum, in Ptf1a::cre;Robo3^lox/lox^ mice 67% of inferior olive neurons have ipsilateral projections [280]. For the flocculus of Ptf1a::cre;Robo3^lox/lox^ mice, 76% of olivary neurons providing climbing fibers were located ipsilaterally [281]. On the other hand, lateralization of mossy fiber projections into the cerebellum and the size, structure and cytoarchitecture of the cerebellum were not affected [280, 281]. Nevertheless, these mice suffer from severe ataxia starting around postnatal day 10 and the motor deficit has been shown to be even more severe than that seen in Lurcher mice, which completely lack cerebellar cortex output [280]. Ptf1a::cre;Robo3^lox/lox^ mice showed deficits in the gain and phase of the optokinetic reflex and vestibulo-ocular reflex in light and higher variability of both reflexes compared with control mice [281].

## Research applications for mutant ataxic mice

### Functional abnormalities

Cerebellar mutant mice suffer from more or less severe cerebellar ataxia but also display a wide range of interesting and variable cognitive impairments and behavioral abnormalities. Therefore cerebellar mutants could provide information about the involvement of the cerebellum in cognitive, affective and executive processes.

Experiments in cerebellar mutant mice have confirmed the role of the cerebellum in motor learning (for review see [282]). Nevertheless, motor learning deficits are not equal in all cerebellar mutants. E.g. as shown by Lalonde et al. [31], Staggerer mice did not improve their motor performance during repeated training, while Lurcher and hot-foot mice did. Eyelid conditioning seems to be a good model of associative learning that enables analysis of the execution of a learned motor response. Using this technique, changes in classical conditioning, in execution of the conditioned response and the modulating role of interpositus and red nuclei were described in Lurcher mice [42, 46, 283]. Pcd mice have also been shown to be partially defective in eyelid conditioning and residual learning ability has been attributed to the deep cerebellar nuclei [83, 284]. Classical eye blink conditioning deficit has also been found in global and Purkinje cell-specific Fmr1 gene knockout mice which show morphological and behavioral features similar to fragile X syndrome patients [285]. Defects of conditioned eye blink responses have also been found in human cerebellar patients [286, 287].

Spatial learning or orientation deficits in various types of mazes have been reported in many types of cerebella mutants: Lurcher [41–44], Hot-foot [62], Pcd [81], Nervous [94], Staggerer [117], Weaver [144], Reeler [192], Scrambler [211] and in Niemann-Pick C disease mouse model [262, 263]; however, difficulties in solving water maze tasks are not uniform (compare references e.g. [44, 81] and [94]). While impaired navigation to a hidden goal with preserved ability to navigate toward a visible goal (pcd, Nervous) is supposed to indicate a spatial learning defect, impaired navigation to both hidden and visible goals (Lurcher) suggests more complex problems [44]. Furthermore, it is not always clear whether the poor performance in the maze is specifically a consequence of impaired spatial learning ability since motor and oculomotor deficits or affective function abnormalities could influence the behavior of the mice in the maze.

Some behavioral abnormalities of cerebellar mutants resemble features of human psychiatric disorders. Conversely, abnormalities in cerebellar morphology have been described e.g. in autistic patients [288–290]. Cerebellar pathology is considered to play a key role in the pathogenesis of autistic spectrum disorders (for review see [291]). Autism spectrum disorders have often been shown to be accompanied by developmental Purkinje cell loss (e.g. [292]) and some patient features are analogous to behavioral and cognitive abnormalities in cerebellar mutant mice [53, 293]. Heterozygous Reeler mice have been proposed, with some objections, as model of schizophrenia or neurodevelopmental psychiatric diseases in general [199–201, 294]. Lurcher mice with their behavioral disinhibition and stress axis hyper-reactivity also mimic some psychiatric abnormalities [42, 49–52].

It is, however, impossible to consider common cerebellar mutant mice to be direct models of psychiatric disorders. For psychiatric disorders involving a cerebellar pathology, there are more specific models, such as Fmr1 gene knockout mice for fragile X syndrome [285] or neuroligin-3 knockout mice, a model for nonsyndromic autism [295]. Nevertheless, detailed analysis of behavioral abnormalities and their correlation with structural, ultrastructural or biochemical changes in the cerebellum of various types of ataxic mutant mice could reveal the role of the cerebellum in control of behavior and the impact of cerebellar defects in behavioral pathology.

On the cell function level, firing patterns of Purkinje cells tuned by the activity of other cerebellar components and cerebellar inputs is crucial for cerebellar functions (for review see [296]). Many mutations lead to a change in spatiotemporal firing patterns and motor and cognitive impairments [296], e.g. CACNA1A gene mutation in Tottering mice [245], *Grik* mutation in Weaver mice [137], mice selectively lacking large-conductance voltage- and calcium-activated potassium channels in the Purkinje cells [297], mice deficient in calretinin [298], etc. In calretinin deficient mice, motor coordination was restored with selective re-expression of calretinin in granule cells [299]. Some mutations led specifically to impairment of plasticity of certain cerebellar neuronal types and their synapses with impact on motor learning (for review see [300]).

### Neurotransplantation research in cerebellar mutant mice

Cerebellar mutant mice are used as investigative models of neurotransplantation therapy for cerebellar degeneration. Pcd and Lurcher mice have been the most frequently used. Recently, however, transgenic mouse models of human diseases have been viewed with increasing interest. Neurotransplantation research not only helps in the search for therapeutic strategies, but also offers deeper understanding of the role of neurogenic (either positive or negative) signals from normal and injured cerebellar tissue. Generally, cerebellar tissue is considered to have low neurogenic capacity.

Pcd mice have been shown to be a good model for neurotransplantation research. Donor Purkinje cells were able to leave solid embryonic cerebellar grafts, migrate to their final position in the molecular layer [301] and become integrated synaptically within the pcd cerebellar cortex [302, 303]. Sotelo and Alvarado-Mallart [301] suggested that the deficient pcd molecular layer exerts a selective neurotropic effect on neurons of the missing category. Re-establishment of corticonuclear projections has also been demonstrated in pcd mice [304–306]. Nevertheless, proximity of the grafted Purkinje cells to the deep cerebellar nuclei is necessary [306] since the granular layer acts as a barrier that prevents nerve fibers sprouting toward the deep cerebellar nuclei [307]. Carletti and Rossi [308] found, that the pcd cerebellum provides signals inducing selective mechanisms that favor the survival of donor Purkinje cells. Bilateral transplantation of a fetal cerebellar cell suspension into the deep cerebellar nuclei led to an improvement of motor performance in pcd mice [309, 310].

Lurcher mice have also been used to study transplantation of embryonic cerebellar cells. Aggregates of grafted cells with organotypic organization on the surface of the host cerebellum and invasion of grafted cells into the host’s molecular layer have been observed [311–313]. Solid embryonic cerebellar grafts survived well for 6 months in both Lurcher and wild type mice but lacked sufficient interactions with the host cerebellum [314]. Timely transplantation of normal embryonic Purkinje cells can prevent degeneration of cerebellar granule cells and inferior olive neurons and this provides evidence for secondary nature of degeneration in these cell categories [312]. A significant improvement in performance on motor tests was observed by Jones et al. [315] after transplantation of mesenchymal stem cells into the cerebellum of newborn Lurcher mice. Donor cells were located adjacent to the Purkinje cell layer and produced neurotrophic factors (BDNF, NT-3 and GDNF) that increased Purkinje cell survival [315].

In newborn nervous mice, intracerebellar transplantation of undifferentiated neural stem cells supported mitochondrial function, rescued Purkinje cells and improved motor coordination [316]. Neural stem cells almost normalized previously elevated levels of tissue plasminogen activator, in the cerebellum, and thereby modulated the pathway associated with Purkinje cell degeneration [316]. In Weaver mice, cerebellar grafts developed a trilaminar organization, grafted granule-like cells proliferated and were able to make synaptic contacts [317].

In SCA1 mice, transplantation of an embryonic cerebellar cell suspension led to improvement in motor function [318]. Transplantation of neural precursor cells improved motor skills, enhanced survival of Purkinje cells and normalized Purkinje cell membrane potentials in SCA1 mice; all this despite none of the grafted cells being able to adopt Purkinje cell-like characteristics [319]. Furthermore, intrathecal injection of mesenchymal stem cells mitigated cerebellar disorganization, suppressed atrophy of Purkinje cell dendrites and normalized deficits in motor coordination in SCA1 mice [320]. In SCA2 transgenic mice, Chang et al. [321] found that intravenous injection of human mesenchymal stem cells increased the survival of host Purkinje cells, delayed onset of disease and improved motor function.

In the mouse model of Niemann-Pick disease type C, mesenchymal stem cells were successfully grafted. Bone marrow-derived mesenchymal stem cell transplantation reduced astrocytic and microglial activation in the cerebellum [267], led to an increase in Purkinje cell numbers and motor skill improvement and it has been shown that electrically active Purkinje neurons originated from existing Purkinje cells through fusion-like events with grafted mesenchymal stem cells [268]. Also transplantation of adipose tissue-derived stem cells rescued Purkinje neurons, alleviated inflammatory responses and restored motor coordination in Niemann-Pick disease type C mice [269].

The first clinical trials have already tested neurotransplantation therapy in human patients with cerebellar degeneration [322, 323]. The results seem to be promising, but cerebellar transplantation is not routine therapy, yet, and intensive preclinical research is still necessary. With regard to the variability in the nature of human hereditary cerebellar ataxias, different approaches for individual diseases will be probably necessary.

## Conclusion

Studies on cerebellar mutant mice have provided a lot of information about cerebellar function, manifestation of cerebellar disorders, and pathogenesis and therapy of cerebellar degeneration. Since Purkinje cell axons are the sole output of the cerebellar cortex, mice with complete loss of the Purkinje cells (e.g. Lurcher, pcd) represent models of functional cerebellar decortication.

Studies of cerebellar degeneration in mutant mice have revealed the mechanisms of cell death, which have been found to be necrosis, apoptosis, or autophagy and it has been even suggested that cell death may involve combinations of multiple pathways [14, 23]. Although the degeneration affects the vast majority of Purkinje cells, in certain of the cerebellar mutants, a few Purkinje cells often survive until late adulthood. The distribution of these cells, as well as the irregular progress of degeneration within the cerebellar cortex often follows characteristic patterns that vary from one mutant strain to the next. This variable Purkinje cells sensitivity to the effects of the mutations shows their heterogeneity [324]. Cerebellar mutant mice are also a model of secondary cell degeneration caused by deprivation of postsynaptic targets or synaptic inputs.

Cerebellar mutants are variable relative to the extent of degeneration of individual cerebellar and extra-cerebellar cell types, and cell death pathways. For example, Lurcher, Purkinje cell degeneration and Nervous mice suffer from severe Purkinje cell loss. In Staggerer or Weaver mutants, the main problem is depletion of granule cells. Poor innervation of Purkinje cells is the dominant pathology in Hotfoot mice, whereas in Reeler, Scrambler and Weaver mice, defective neuronal migration is responsible for cerebellar disorganization.

Though, the basic manifestation of cerebellar dysfunction is similar in most of the mouse models of cerebellar degenerations, some particular signs differ. Moreover, some of the mutations have extra-cerebellar impacts, which are integral components of the phenotype (e.g. olfactory bulb, retina and thalamus degeneration in pcd mice, dorsal cochlear nuclei of Nervous mice, complex CNS disorganization in Weaver and Reeler mice – see above). Such variability and complexity of manifestations in ataxic mutant mouse models is advantageous regarding the complexity of symptoms of human cerebellar degenerations. They provide tools to search for factors determining particular phenotypic features and relationships between morphologic and functional abnormalities. Knowledge of the factors modifying the course of the disease is important for understanding its pathogenesis as well as for selection of optimal therapeutic approaches. On the other hand, interpretation of examinations of particular neural functions in cerebellar mutant mice is difficult and ambiguous since the performance on tests can be influenced by multiple factors such as motor abilities, cognitive functions, anxiety or motivation.

It should be taken into account, however, that mouse models of hereditary cerebellar degenerations have serious limitations that prevent direct translation of findings to humans. There are, of course, species differences in anatomy, metabolism, behavior, etc. between mice and men. Spontaneous mouse mutations are usually not identical to human ones and therefore mouse diseases can only be similar to human diseases. On the other hand, transgenic mice carry human pathological alleles and thus they can be used as models for specific human diseases. Nevertheless, even in transgenic mice the action of the mutation could significantly differ from the natural human mutation. The transgene could be under control of different promoters and its expression could differ from that seen in humans regarding intensity and cell type. The transgene usually does not replace the mouse wild type allele, if not knocked-out. In these cases the mouse has both pathological as well as fully expressed normal gene products. Autosomal dominant ataxias appear in humans mostly in the form of heterozygotes. However, mice can also be studied as homozygous individuals. These experiments have shown that homozygous mice often display more severe pathological phenotype than heterozygous mice suggesting that the diseases might be more accurately described as having a semi-dominant nature. Finally, cerebellar mutant mice have the phenotypic traits of the original strain, which could also interfere with the manifestation of the mutation of interest. Therefore it is not easy to compare two cerebellar mutants when they are derived from different mouse strains. Some mutants are available in more strains of origin and some caution in necessary when comparing findings of studies that have used different strains of the same mutant. Most transgenic mice are created using F1 hybrid strains which are not stable across subsequent generations. Despite the limitations, cerebellar mutant mice are invaluable tools for research, when the goal is a better understanding the pathogenesis of cerebellar degenerative disorders and, hopefully, finding effective therapies for humans.

Cerebellar mutant mice will continue to serve as valuable tools in preclinical studies investigating therapeutic methods for treating human cerebellar degenerations. Nevertheless, deep phenotypic characterization, especially of the new transgenic mouse models, and elucidation of the pathogenesis and relationship of the functional disorders to the cerebellum will remain important. Verification of the conformity of the mouse models with human diseases on the morphological, functional and molecular level is also crucial for translation of experimental research to human medicine.
